# How Adaptive TNCs Can Improve Transportation Quality for Older Adults and Stretch Resources for Smaller Communities

**DOI:** 10.1093/geroni/igaf122.1294

**Published:** 2025-12-31

**Authors:** Ron Brooks, Steve Cummings, Susan Starwalt

**Affiliations:** UZURV, Richmond, Virginia, United States; UZURV, Richmond, Virginia, United States; UZURV, Richmond, Virginia, United States

## Abstract

Established in 2017, UZURV is an adaptive Transportation Network Company (TNC) aimed at enhancing mobility services for older adults and individuals with disabilities. It collaborates with public transit agencies, social service organizations, and communities to deliver millions of door-to-door trips annually across approximately 25 communities in 15 U.S. states. UZURV’s innovative model involves tailoring its technology, driver onboarding processes, and service methods to address specific community needs. This has set a standard in the rideshare industry, paving the way for other providers to emulate this approach by adjusting to the unique service demands and regulatory requirements of smaller communities. This presentation explores UZURV’s adaptations of the rideshare model to meet the needs of those requiring door-to-door service, including wheelchair accessibility, service animal accommodations, and options for people who do not have, or cannot use, smartphones. We will address how UZURV aligns its operations with agency contractual and compliance mandates, detailing the implementation of background checks, employee training, and substance abuse testing and reporting. Furthermore, this presentation will illustrate how compliant rideshare providers like UZURV can augment traditional community transport systems and enhance service coordination. We will present operational and financial data demonstrating that rideshare services can offer safe, accessible, and dignified transportation at lower costs compared to traditional paratransit options. This evidence will highlight the viability of the rideshare model in expanding transit opportunities for underserved populations while maintaining rigorous safety and accessibility standards.

